# Police Fitness: An International Perspective on Current and Future Challenges

**DOI:** 10.3390/sports13070219

**Published:** 2025-07-07

**Authors:** Robin Orr, Elisa F. D. Canetti, Suzanne Gough, Kirstin Macdonald, Joe Dulla, Robert G. Lockie, J. Jay Dawes, Sam D. Blacker, Gemma S. Milligan, Ben Schram

**Affiliations:** 1 Tactical Research Unit, Bond University, Gold Coast 4226, Australia; ecanetti@bond.edu.au (E.F.D.C.); sgough@bond.edu.au (S.G.); kmacdona@bond.edu.au (K.M.); jdulla@georgiasouthern.edu (J.D.); bschram@bond.edu.au (B.S.); 2 Faculty of Health Sciences and Medicine, Bond University, Gold Coast 4226, Australia; 3 Tactical Athlete Project, Georgia Southern University, Statesboro, GA 30458, USA; 4 Center for Sport Performance, Department of Kinesiology, California State University, Fullerton, CA 90840, USA; rlockie@fullerton.edu; 5 Oklahoma State University Tactical Fitness and Nutrition Lab, Oklahoma State University, Stillwater, OK 74078, USA; jay.dawes@okstate.edu; 6 Occupational Performance Research Group, Institute of Applied Sciences, University of Chichester, West Sussex PO19 6PE, UK; s.blacker@chi.ac.uk; 7 School of Psychology, Sport and Health Science, University of Portsmouth, Portsmouth PO1 2ER, UK; gemma.milligan@port.ac.uk

**Keywords:** law enforcement, physical fitness, occupational fitness, recruitment, capability

## Abstract

Poor officer fitness can lead to decreased occupational task performance, injuries, increased absenteeism, and a variety of negative health sequalae further adding to the challenges of staffing law enforcement agencies. Optimizing the physical fitness for both serving officers and new recruits is critical as their loss is, and will increasingly be, difficult to replace. However, maintaining and recruiting a physically fit workforce faces several challenges. For serving officers, shiftwork is known to decrease motivation to exercise and negatively impact sleep and diet. Additional factors impacting their fitness includes age-related declines in fitness, increasing obesity, long periods of sedentarism, and negative COVID-19 effects. Concurrently, recruiting physically fit recruits is challenged by declining levels of fitness, reduced physical activity, and increasing obesity in community youth. Ability-based training (ABT), individualizing physical conditioning training based on the existing fitness levels of individuals within a group, offers a potential solution for delivering physical conditioning to groups of applicants, recruits, and officers with a range of physical fitness capabilities. Law enforcement agencies should consider implementing ABT during academy training and ongoing fitness maintenance to minimize injury risk and optimize task performance.

## 1. Introduction

Policing is an inherently dangerous and physically demanding occupation, which requires officers to put themselves at risk, often on a daily basis [1]. The nature of policing varies between nations [2]; throughout this review, information from studies conducted with a range of police agencies is reported to describe general considerations relating to physical fitness and health in these populations. General policing job tasks can range from checking a person’s identity to attending domestic violence situations or vehicle accidents [3]. Daily role-related physical requirements can include, but are not limited to, long periods of sitting in a vehicle while being exposed to whole-body vibration, long periods of stationary standing or roving foot patrols, pursuing offenders on foot, grappling with offenders, and pushing, pulling, and dragging those resisting [4,5,6]. All these tasks are typically performed while wearing and carrying loads of around 10 kg [7] during shift lengths ranging from eight to twelve hours for single shifts [8] (doubling to sixteen hours or more for back to back shifts [9]), across their careers. The inherent dangers of the job, the potential need to use force, and the general physicality that may be associated with their duties highlight the need for officers to remain physically fit [1].

The nature of policing brings with it several inherent occupational risks to officer fitness and health. Factors that include sedentarism [10], poor nutritional options [11], and shiftwork [12] all contribute to negatively impact the police officer, with the latter being of particular concern [13,14]. Shiftwork has been found to impact on the body’s circadian rhythms, leading to sleep and circadian rhythm disruption (SCRD) [15]. Apart from impacting job performance [6,16,17], SCRD can lead to various general health concerns, including cardiovascular disease (CVD) and, potentially, carcinogenesis [18,19]. Furthermore, shiftwork has been found to have a negative impact on the desire to conduct physical activity [20,21], further increasing sedentarism and fitness loss, as well as excessive calorie consumption from snacks [22], factors which in turn compound adverse health outcomes (e.g., obesity [22]). Regarding fitness loss, Orr et al. [23] found that police officers were generally less fit than age-matched police recruits. Lockie et al. [24] reported that officers who had served for 48+ months were less fit than officers who had served for 24 to 47 months, who themselves were less fit than officers who had served for less than 24 months, with the latter officers in turn less fit than recruits. In a longitudinal study of officers from the same agency, Lockie et al. [25] found that officers who had completed their first duty assignment in custody were less fit than they were when completing their recruit training, taking longer to complete their occupationally relevant Work Sample Test Battery.

Given factors like SCRD and the loss of fitness impacting officer health, it is not surprising that police officers are twice as likely to suffer from CVD when compared to the general population [26]. Furthermore, police officers have a significantly higher mortality rate than the general population, with male police officers in the 50–54-year-old category having a 40% probability of death compared to only a 1% probability for commensurate males in the US population [22]. Loss of fitness in particular brings with it notable concerns given the potential sudden requirement to perform tasks at near maximal efforts and that most cardiovascular incidents are considered to happen during or within 24 h following physical exertion [27]. In arrest-related deaths in custody, research suggests that officer deaths were usually preceded by significant physical exertion, being restraint with physical resistance followed by fleeing on foot [28]. Additional occupational risks associated with the reduced life expectancy of officers includes shift work, posttraumatic stress disorder, and stress as well as potential environmental exposures (e.g., chemicals) [22,29]. Physical exercise and fitness are known to mitigate and help manage some of these risks, notably shift work [30], PTSD [31,32], stress, anxiety, and other physical and mental health challenges [22,33].

Fitness is critical to policing capability beyond just protection from adverse health consequences [34]. During academy training, the level of fitness of trainees has been found to be associated with training success in law enforcement [35,36,37], with these findings being in line with other tactical populations including the military [38,39] and firefighters [40]. For qualified officers, fitness has been found to be associated with a variety of occupational tasks, from dragging a person and vaulting over a fence [41] to chasing an offender on foot [42], use-of-force ability [43], and marksmanship performance [44]. The importance of fitness in generating, maintaining, and optimizing the police workforce is clear. However, several factors continue to impact the fitness of this population, which in turn affects the future of maintaining an operational police force. The aim of this narrative review is to summarize the current and future challenges to police physical fitness and discuss a potential mitigation strategy. The review is structured around three central themes being currently serving law enforcement personnel, future serving populations (populations from which future officers will be drawn), and the use of ability-based training as one potential solution.

## 2. Current Serving Law Enforcement Populations

For a variety of reasons, ranging from fewer recruits (discussed in the next section) to increasing rates of resignations and retirement [45,46,47], many agencies have, or are at risk of having, a large number of vacancies, with the number of serving officers continuing to decline [48,49]. For example, in Australia, across its seven states and territories, police force vacancies are reported to be up to 12% [50]. In the USA, law enforcement agencies are noted as presenting with an average staffing deficit of nearly 10%, with some agencies reporting retirement eligible staff making up 24% of the agency’s workforce [49]. These vacancy rates ultimately mean that those police officers who are currently serving are taking on additional work to maintain agency capability and are thus increasing their occupational exposure (e.g., more shift work, more occupational load carriage, etc.). With the aforementioned level of understaffing, maintaining the currently serving police workforce becomes imperative. However, more so than just maintaining the workforce, ensuring the remaining officers are fit enough to perform their required job tasks is critically important. Unfortunately, maintaining serving officer fitness faces several challenges, from an aging police force to increasing levels of obesity and poor health associated with aging, lifestyle, and occupational requirements.

### 2.1. Fitness in an Aging Workforce

Research suggests that the average age of currently serving police agency officers is increasing [51]. As an example, Smith [51] highlights how over the last 30 years, the average age of a police officer has risen from 36 years of age to 41 years of age. In the US, more than 50% of police officers are over 40 years of age [52]. For police agencies, this aging workforce brings increased occupational experience, but also a decline in fitness associated with aging [53] as well as the loss of fitness associated with the nature of police service [23,54]. In addition, injury rates in law enforcement are three [55] to four [56] times higher than the general population with officers suffering as many as 2.5 injuries per officer per year [57]. As such, these older officers who have longer occupational lifespans are likely to have sustained numerous injuries during their service, which can not only reduce their fitness over time [58] but increase their risk of future injury [59,60].

### 2.2. Obesity and Health in an Aging Workforce

Increasing rates of obesity across the adult population continue to be a challenge with an increasing number of middle- and high-income countries confronted with an epidemic of severe obesity that, far from plateauing, appears to be escalating [61]. Concomitant to this increase in obesity come comorbid factors including metabolic syndrome and Type II diabetes, with the latter having more than tripled between 1990 and 2019 [61]. In policing populations, research suggests that 42% [62] to 65% [63] of police officers are obese and at risk of CVD [64]. This concern is highlighted with the mean age of officer deaths in the U.S. due to circulatory causes being 46.3 years [55], only slightly above the mean age of officers serving in many agencies [51].

Apart from CVD risk, high levels of obesity are negatively associated with workplace absenteeism following injury [65], impacting law enforcement productivity and further depleting an understaffed workforce. Higher levels of obesity may also negatively impact officer task performance. For example, Dicks et al. [66] found that the percentage of body fat was significantly associated with the time taken to complete a Physical Readiness Assessment, predicting 26% of the variance in a regression model. As such, higher levels of obesity have the potential to reduce the ability of some officers to perform their duties while concomitantly, through increased absenteeism, increasing the work demands (and exposures) of fellow officers.

### 2.3. COVID-19 Effects on a Serving Population

During the COVID-19 pandemic, police officers were a high-risk population for COVID-19 exposure. Baker et al. [67] noted that around 52% of personnel serving in protective service occupations were exposed to COVID-19 at least once per month and that 29% were exposed more than once per week. Thus, it was not surprising that COVID-19 infection rates were three times higher in first responders when compared to the general population [68]. Work by Schwendinger and colleagues [69] highlights how, even long-term post infection (several months), the aerobic fitness of adults may still be below 90% of predicted values. Of most importance, these authors noted that deconditioning was not the primary reason for this reduced aerobic fitness and that the post COVID-19 sequelae was multifaceted, requiring medical diagnosis and treatment. Simply, more physical conditioning alone will not mitigate this loss of fitness. Of note, one factor influencing recovery from COVID-19 post infection is the severity of the infection of the individual. Those community-recovered patients (i.e., those not requiring any form of hospitalization) in the medium term (5 months) have been found to be no different to general healthy adult controls in relation to fitness [70]. In February 2025, the World Health Organization reported that approximately 6 in 100 people develop serious long-term effects, which are referred to as post-COVID-19 or Long COVID [71]. Long COVID is characterized by a range of symptoms that commence within 12 weeks of the initial COVID-19 onset, including fatigue, breathlessness, muscle or joint pain, or impaired sleep [71].

Apart from experiencing symptoms of COVID-19, and potentially Long COVID, the stay-at-home requirements caused by lockdowns may have exacerbated sedentary behavior and low physical activity in adults [72,73], with these behaviors still present post pandemic [74]. This increase in sedentary behavior in adults, by around 2 h per day [72], brings with it known concerns associated with low levels of physical activity, including a further loss of fitness [75] and increases in obesity [76]. Anaerobic and aerobic fitness have been found to be significantly lower post pandemic in both males and females, with reductions in explosive power and upper body strength also observed in females [75]. These losses are notable given that they are fitness measures associated with common police fitness test performance [77]. Additionally, the rise in body mass, which negatively affects the fitness performance of police personnel, has been exacerbated by the increased sedentary behavior during lockdowns. This has contributed to a global surge in obesity rates, now referred to as “covibesity” [76]. Thus, the COVID-19 pandemic has, either directly (i.e., infection) or indirectly (i.e., lockdowns and reduced physical activity) negatively impacted the level of fitness and rates of obesity of currently serving officers, increasing the challenge of ensuring officers are fit for duty and at a lower risk of injury and mitigating poor health outcomes. Whilst Long COVID symptoms often subside after 4–9 months, global estimates indicate 15 in 100 people have ongoing symptoms 12 months post diagnosis [71]. There is a paucity of research and limited large-scale studies to determine the most clinically effective treatment of Long COVID [71].

## 3. Future Serving Populations

Noting the increasing officer exiting and retirement rates [45,46] and current large number of vacancies [48], replacing officers is a critical requirement. These replacements are typically drawn from younger adults from the community. Unfortunately, the pool of younger adults is decreasing with many countries suffering declining births rates to levels below that required to maintain their population (i.e., ~2.1 children per woman [78,79]). Adding to these challenges, those young adults who make up the diminishing recruitment pool generally have lower levels of fitness [80], are more sedentary [72], and have higher rates of obesity [81]—with all these factors further negatively impacted by COVID-19 [82].

### 3.1. Fitness in Youth

The importance of aerobic fitness in injury mitigation for new trainees has been well established [83,84,85]. Research by Tomkinson et al. [86] investigating trends in the aerobic fitness of children and adolescents aged 9–17 years across 19 countries reported a decrease in aerobic fitness of 7.3% over a 33-year period (1981–2014), equivalent to approximately 1% per year. This decline in aerobic fitness was larger for boys (−1.3% per decade) than for girls (−0.7 per decade) [86]. The authors postulated that this decline in aerobic fitness could be attributed to various factors including an increase in body mass index (BMI), a decline in physical activity, or an increase in sedentary behavior [86]. Further work by Tomkinson et al. [80], investigating various running assessments (e.g., 20 m shuttle run, 1.2 km and 1.6 km distance runs across 55 countries), found a constant decline in running speed in both boys (−0.46% per year) and girls (−0.41% per year), over the same 20-year period (1980–2000). The authors postulated that decreased physical activity more so than increased BMI was the primary factor for the decrease in running speed; noting that BMI likewise increased (0.6% per year [87]) over that period. As such, younger adults progressing to complete law enforcement fitness assessments that involve running speed, being most assessment batteries [77], are less likely to meet a given fitness standard if standards have not been updated or are required to remain extant (e.g., prediction of injury risk during training) [88].

In addition to lower levels of aerobic fitness, research suggests that younger adults possess lower levels of grip strength [89]. In their 2016 study, Fain and Weatherford [89] compared the grip strength of 20–24 year old males to those presented in 2002, finding that, on average, right-hand grip strength was 9.1 kg lower and left-hand strength was 2.6 kg lower with a similar trend amongst female groups. Given that grip strength is associated with tasks like body or casualty drags [90] and influences use-of-force outcomes [91], as well as marksmanship [91] in police recruits, the decline in grip strength presents as a notable challenge to new recruits entering policing roles.

### 3.2. Sedentarism and Physical Activity in Youth

In general, youth spend a considering amount of time being sedentary, equating to two thirds of their day, which is typically greater during school days than non-school days [92]. This loss of physical activity in schools is noted as starting within the child’s first school year [93]. In an observation of Grade 1 school children, Macdonald et al. [93], noted that 86% of 5305 observations (30 s time intervals) during school class time were considered sedentary, with only 2% of observed time reaching moderate-to-vigorous physical activity. Further adding to this concern is the increase in sedentarism that comes with increased screen time (inclusive of computer use, social media, video gaming, etc.) [94,95] with 17-year-olds who engage in videogaming for more than 3 h per day, as an example, found to have lower levels of physical fitness, poorer eating habits, and increased incidence of obesity [96]. Of concern, research suggests that this sedentarism has potentially increased further following the COVID-19 pandemic [72,97]. As noted above, it is this increased sedentarism and concomitant decrease in physical activity and increase in BMI that is the leading contributor to fitness loss in youths [86,87]. While this loss of fitness is of concern, the reduction in physical activity brings with it additional negative impacts, like the loss of motor competency [98]. Motor competency (‘movement literacy’), which is considered proficiency across a wide range of motor skills and associated underlying mechanisms [99], has likewise been suggested to have declined in youth [98]. Thus, increased sedentarism and reduced levels of physical activity raise concerns for future serving populations., with these potential candidates having not only less fitness but also poorer motor skills and physical development in general [98,100].

Noting that the relationship between motor competency and physical activity is complex and not yet fully understood [101], research suggests that children who are actively engaged in sport are specializing in that sport at a younger age [102,103]. As such, they are staying with the one sport across their years of participation, rather than participating in a ‘fall’ and then ‘spring’ sport [102]. This in turn may restrict their general athleticism to the specific set of skills associated with their sport rather than a more global range of skills. In addition to a loss of general motor competency, the higher sports-specific repetitions can increase their risk of sustaining an overuse injury at a younger age [102,103], placing them in a ‘high injury’ risk group for future injuries [104,105].

### 3.3. Obesity and Health in Youth

With obesity being a growing international problem [106], research suggests than one in 3–4 children in the United States of America [107], the United Kingdom [108], and Australia [109], as examples, are overweight or obese. In children, obesity is known to come with a range of comorbidities, including metabolic disorders, cardiac hypertension, and coronary heart disease [106]. Given the research identifying cardiometabolic concerns associated with the law enforcement profession [26,64], these comorbid conditions raise concerns. Additional comorbidities in children associated with obesity include depression, anxiety, and sleep disorders [106]; factors also associated with shiftwork and SCRD, which are in turn associated with the nature of police work. Thus, notable concerns are raised if new recruits enter the law enforcement profession with conditions that are known to be exacerbated by the nature of the occupation.

### 3.4. COVID-19 Effects on a Future Serving Population

The COVID-19 pandemic saw many countries enter periods of lockdown, limiting the ability of children to participate in both formal (i.e., physical education lessons, sports, etc.) and informal (i.e., game play during school recess or after school, bike riding, etc.) physical activity [82,110,111]. As such, the trend of decreasing physical activity in youth was exacerbated by this lack of activity [110,111]. Downstream impacts of these lockdowns and reduced activity lead to further declines in motor competency and physical fitness and increases in obesity. As an example, research by Wahl-Alexander et al. [82] of 131 male and 133 female US school children in Year 3 through Year 8 found that not only was there a significant increase (+10%) in students’ BMIs over the duration of a COVID school year, but so too were there significant decreases in aerobic (−27%) and muscular endurance (pushups = −37%, sit ups = −20%) [82]. In regard to obesity, a study investigating obesity trends in 2–19 year olds from the US following the COVID pandemic reported an increase in obesity from 19.3% in August 2019 to 22.4% in August 2020 [112]. In the United Kingdom, the number of Year 4 and 5 children classified as obese or severely obese rose from 33% (October 2019) to 47% (November/December 2020) following the pandemic [113]. A recent retrospective cohort study indicated that compared to the pre-pandemic period, excess increases in obesity of both boys and girls persisted over 3 years, with excessive underweight and poor visual acuity amongst boys [114]. As such, with this demographic providing the source of future recruits for law enforcement, this further exacerbation of a variety of already disconcerting factors due to COVID-19 (i.e., decreased activity levels, physical fitness, and movement competency and increased obesity) is of concern.

Increased sedentarism and obesity, reduced physical activity, lower levels of fitness, poorer movement literacy, and post-COVID-19 impacts all contribute to challenges in fitness development and maintenance in cohorts who present with diverse levels of fitness and movement skills, be they incoming recruits or currently serving officers. This challenge is further exacerbated by the increasing need to mitigate injuries so as not to lose potential recruits or officers and maintain the workforce.

## 4. Ability-Based Training

A one-size-fits all physical conditioning approach is common in law enforcement [36,115,116,117,118]. Examples include cohorts all running the same distance, often in a group, performing the same exercises during a circuit (e.g., back squat with a 10 kg torsion bar), etc. One concern with this training approach is the loss of fitness in fitter personnel and increased injury risk in less fit personnel, as the training stimulus is often directed toward the mean capability of the group. The impact of this approach is seen in the study by Moreno et al. [119], whereby less fit recruits were found to be working harder in two different circuits when compared to the fitter recruits. In their study, recovery hears rates, as measured by the YMCA Step Test, were significantly lower in the recruits in the top 25% of fitness as opposed to those in the bottom 25% of fitness (113.60 ± 4.39 versus 156.00 ± 5.52, *p* < 0.05). Furthermore, a one-size-fits-all training approach may mean that the majority of female recruits, who generally present with a lower level of aerobic fitness, will be working at a relatively higher intensity for the same exercise when compared to male recruits, thereby increasing their risk of injury [120].

Ability-based training (ABT) presents as a viable and evidence-based alternative to the one-size-fits all approach. ABT allows for a physical conditioning program to be tailored to the ability level of individuals within a group [115,119,121,122,123]. This approach to training was first formally noted in the study of Knapik et al. [121], in their work with the US military. While a common concern with this training approach is that those who are less fit will not meet training requirements, Orr et al. [115] found no differences in fitness performance on a Shuttle Run between police recruits that completed an ABT running program versus those that completed the traditional group running approach, both consisting of eight training sessions. However, not only were recruits who completed the ABT program at a lower relative risk of injury (RR = 0.31–0.59, p = 0.24–0.28) but the physical conditioning session time was reduced (−15 min), allowing recruits completing the ABT program to undertake movement skills sessions. Furthermore, whereas the ABT group commenced the training at a mean level of aerobic fitness below the control group (60.98 ± 16.45 versus 63.32 ± 15.7 shuttles), they completed their training at a mean level above that of the control group (70.11 ± 16.54 versus 67.48 ± 15.95 shuttles). One note of caution, prior to providing examples of how ABT can be practically implemented, is the lack of research using this training approach, with the majority of the limited research conducted in law enforcement agencies [115,119,122,123].

## 5. Practical Application

**Figure 1:**
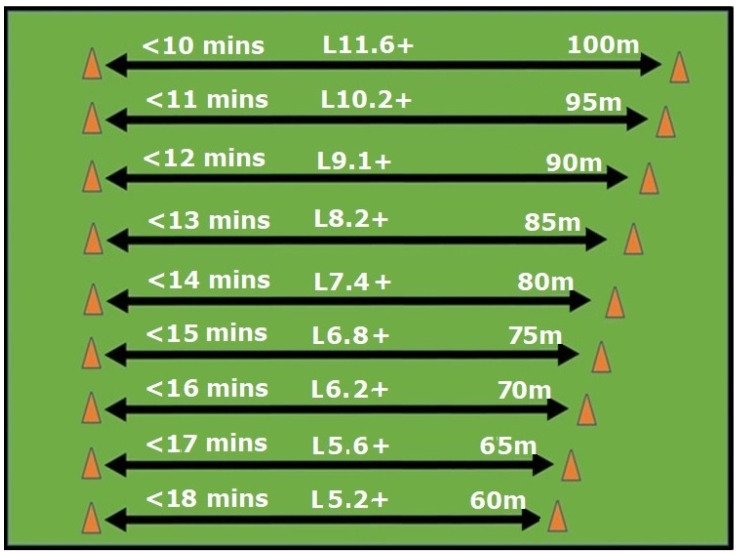
Example of an ability-based training session with distances (in meters) set by 2.4 km (1.5 mi) run times or 20 m Progressive Shuttle Run performance. Further groupings could be added as required.

**Figure 2:**
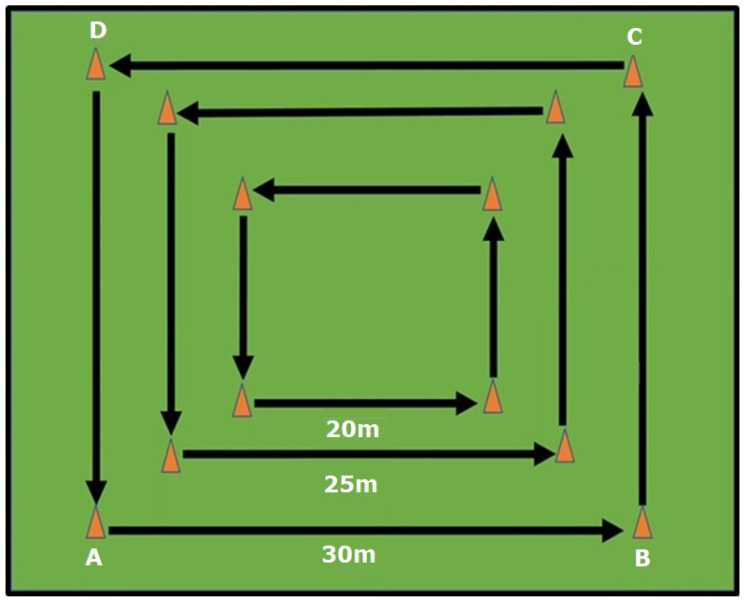
Example of an ability-based training session with distances (in meters) using a box approach.

Examples of ABT approaches for metabolic and neuromuscular conditioning are shown in Figure 1 and Figure 2 and Table 1. Figure 1 demonstrates a means of stratifying metabolic (anaerobic and aerobic) conditioning stimuli to cater to varying fitness levels. In the example (Figure 1) groupings are based either on their most recent 2.4 km (1.5 mile) or 20 m Progressive Shuttle Run performance. In this approach, distances covered are manipulated based on fitness level rather than using a set distance relay for all to complete. A small field or even parking lot could be used, with the distances between cones depending on the metabolic conditioning intent. Groupings behind each cone could be based on rest recovery ratios. For example, each cone could consist of four people, with one running every fourth lap (1:3 rest–recovery ratio). For longer distances or increased intensity, this could be reduced to three people with one running every third lap (1:2 rest–recovery ratio). The benefits of the approach, noted in Figure 1, would have the fittest and least fit all starting and finishing around the same time and at the same point, yet fitter individuals would cover greater distances in the given time.

In the example shown in Figure 2, a box format could be used, with those with lower levels of fitness moving around the inner square while those with greater fitness move around the outer squares, thereby covering a greater distance rather than having all participants running the same distance regardless of fitness. Personnel could run around the entire box at a given pace (e.g., 80% pace around A-B-C-D) or complete fartlek shuttles (e.g., 70% pace A-B and C-D-A, 80% pace B-C). Personnel could run in teams of three completing two legs each (e.g., person 1 runs from A-C, person 2 from C-A, and person 3 from A-C) or in teams of five, with each person completing one leg. Again, distances and number of laps can be manipulated depending on available space (e.g., a nearby park, inside area of an athletic track, parade ground, etc.).

Apart from metabolic conditioning, ABT can also be applied in a circuit format. Baseline could be considered Level 0, with regressions (−levels) or progressions (+levels) applied (See Table 1). In the example provided in Table 1, six fundamental movement patterns are being used (parallel lift, split lift, pull, push, bend, and twist). Initially all personnel can be assessed at Level 0, which would typically be the single exercise given in group circuits (e.g., squats, push-ups). However, the ABT approach includes variations accommodating movement skills and personal ability. For example, a person may be able to progress to a weighted squat (parallel lift Level 2) but regress to a partial-range lunge (split lift Level −2). The versatility and scalability of exercises based on individual capabilities would allow for this type of circuit to be conducted two to three times a week during academy training or at a police station, thereby meeting the American College guidelines for resistance training [124]. Furthermore, these exercises can be varied depending on need and expanded to include occupational tasks like body drags or loaded/farmer carries.

## 6. Conclusions

Physical conditioning can mitigate many of the current and emerging threats to the fitness and health of both current and future officers. However, for conditioning programs to be truly effective, they must adapt to the increasingly diverse needs of the workforce. This includes addressing the challenges faced by serving officers, who are older, may present with a range of injuries, have reduced fitness levels, and are increasingly obese, and by recruits, who have reduced fitness levels, reduced movement skills, and increased levels of obesity. Simply put, a one-size-fits-all approach for police fitness training will not suffice. ABT emerges as one critical solution, offering a flexible framework that can be tailored to accommodate a broad spectrum of physical capabilities in a group setting. By addressing individual needs, this approach not only enhances fitness outcomes but also strengthens the resilience of the police force as a whole. Failure to adopt such adaptive training methods risks exacerbating the existing fitness decline and undermining the long-term effectiveness of policing, jeopardizing both the health of officers and the safety of the communities they serve. Law enforcement agencies should consider implementing ABT during academy training and ongoing fitness maintenance to minimize injury risk and optimize task performance.

## Figures and Tables

**Table 1:** Example of a group physical conditioning circuit with progressions and regressions based on an individual’s ability.

LEVEL	Parallel Lift	Split Lift	Pull	Push	Bend	Twist
	Squat	Lunge	Pull	Push	Trunk Flexion/Extension	Trunk Rotation
LEVEL −4	Assisted Squat	Step Up	Cable Pull	Wall Push Up	Incline Sit Up	Swiss Ball Seated Hip Circling
LEVEL −3	Sit/Stand Squat	Isometric Lunge	Standing Pull Up	Hands Elevated (60 cm)	Eccentric Sit Up	As per −4, add Upper Body Twist
LEVEL −2	Partial Squat	Partial Lunge	Incline Pull Up	Push Ups on Knees	Swiss Ball Sit Up	Swiss Ball Seated Wood Chop
LEVEL −1	Swiss Ball Wall Squat	On-the-spot Lunge	Lying Pull Up	Hands Elevated (30 cm)	Fingertips-to-Knees Sit Up	Kneeling Wood Chop
**LEVEL 0**	**Body Squat**	**Step Lunge**	**Jump Pull Up**	**Push Up on Toes**	**Wrist-to-Knees Sit Up**	**Standing Woodchop**
LEVEL 1	Arms Overhead Squat	Walking Lunge	Bent Leg Band Pull Up	Feel Elevated (30 cm)	Full Sit Up	Loaded Standing Woodchop
LEVEL 2	Weighted Squat	Loaded Lunge	Pull Up	Feet Elevated (60 cm)	Full Sit Up Hands on Head	Med Ball Twist and Pass
LEVEL 3	Arms Overhead Loaded Squat	Directional Lunge	Loaded Pull Up	Loaded Push up	Weighted Full Sit Up	Dynamic Twist and Pass
LEVEL 4	Plyo Squat	Walking Lunge with Contralateral Press or Upright Row	Mobile Pull Up	Plyo Push Up	Full Sit Up to Stand with No Hands	Diagonal Mobile Wood Chop

## Data Availability

All data informing this review is available via the referenced articles.

## Abbreviations

The following abbreviations are used in this manuscript:

ABT	Ability Based Training
BMI	Body Mass Index
CVD	Cardiovascular Disease
